# What is new in the 2018 ESC guidelines for the management of cardiovascular diseases during pregnancy?

**DOI:** 10.1007/s00508-019-1529-y

**Published:** 2019-09-23

**Authors:** Irene M. Lang

**Affiliations:** Department of Internal Medicine II, Division of Cardiology, Medical University of Vienna, Währinger Gürtel 18–20, 1090 Vienna, Austria

**Keywords:** Pregnancy heart team, Aortic disease, Cardiomyopathy, Pulmonary hypertension, Risk stratification

## Abstract

Cardiovascular diseases during pregnancy are the most common causes of pregnancy-associated mortality.

Vaginal delivery is the preferred mode of birth in the majority of pregnancies.

It is recommended that patients with modified World Health Organization (mWHO) class IV risk are counselled against pregnancy.

Patients carrying mWHO II–III, III, and class IV risks should undergo prepregnancy counselling by a multidisciplinary pregnancy heart team to determine a delivery plan and define postpartum care.

Specific medications should not be principally withheld in pregnancy but the risk-benefit ratio should be carefully evaluated prior to administration.

Beta blockers are recommended during and after pregnancy for congenital long QT syndrome and catecholaminergic polymorphic ventricular tachycardia.

Low molecular weight heparin is the ideal substance for prophylaxis and treatment of venous thromboembolism in pregnancy under weekly monitoring of anti-factor Xa activity.

The 2018 European Society of Cardiology (ESC) guidelines for the management of cardiovascular diseases during pregnancy have recently been published in the European Heart Journal [1]. These guidelines were prepared by a multidisciplinary group of experts. Guidelines are intended to provide guidance in physicians’ decision making by a summary of all available evidence at the time of the writing process, including new concepts and treatments as per evidence and experience and by defining standards of organization of care. For example, staffing requirements for the new interdisciplinary pregnancy heart team are provided, conditions are defined where counselling is required, an optimal care setting is defined and minimum follow-up visits during pregnancy including recommended logistics for delivery in relation to calculable risk are described.

The present article presents a brief and condensed summary of new and practically relevant items contained in the 2018 ESC guidelines for the management of cardiovascular diseases during pregnancy [1], highlighting what is new since the last edition of the guidelines in 2011.

## General introduction

Pregnancy is a physiological condition that mimics disease. Therefore, the clinical diagnosis of common conditions that are associated with fluid retention, such as cardiorenal syndromes, may require diagnostic practices that divert from accepted standards of operations. For example, diagnosing heart failure may be more difficult because the physiological changes that occur during pregnancy may mimic cardiovascular disease, and thus mask an underlying disease condition. It is therefore necessary to take the condition of pregnancy into account, to perform in-depth analysis of the medical history, particularly the history of preceding pregnancies, and histories or family histories of hypertension or pre-eclampsia, and to perform a physical examination bearing in mind more subtle signs of disease. Dyspnea that is out of proportion to the pregnancy condition or a new heart murmur necessitate immediate transthoracic echocardiography. It is important to measure blood pressure in a standardized fashion, to search for proteinuria, and repeat those examinations throughout pregnancy.

### The mWHO classification of maternal risk

It is recommended to perform risk assessment in all women of childbearing age with cardiac diseases and before conception, using the mWHO classification of maternal risk in pregnancy [2] (class of recommendation I, evidence level C), (see Table 1). Risk estimation should be further refined by taking into account predictors that have been identified in studies that included large populations of pregnant women with various diseases, such as the cardiac disease in pregnancy (CARPREG) study [3], the ZAHARA study [4, 5] and the registry of pregnancy and cardiac disease (ROPAC) [6]. Fertility treatment is contraindicated in women with mWHO class IV. Fertility treatment requires a careful risk-benefit ratio assessment in women with mWHO class III disease, and in women who need anticoagulation.

**Table 1:** Modified World Health Organization (mWHO) classification of maternal cardiovascular risk (mod. from [1])

	mWHO 1	mWHO II	mWHO II-lll	mWHO III	mWHO IV
Diagnosis (if otherwise well and uncomplicated)	Small or mild – pulmonary stenosis – patent ductus arteriosus – mitral valve prolapse	Unoperated atrial or ventricular septal defect	Mild left ventricular impairment (EF >45%)	Moderate left ventricular impairment (EF 30–50%)	Pulmonary arterial hypertension
			Hypertrophic cardiomyopathy	Previous peripartum cardiomyopathy without any residual left ventricular impairment	Previous peripartum cardiomyopathy with any residual left ventricular impairment
	Successfully repaired simple lesions (atrial or ventricular septal defect, patent ductus arteriosus, anomalous pulmonary venous drainage)	Repaired tetralogy of Fallot	Native or tissue valve disease not considered WHO I or IV (mild mitral stenosis, moderate aortic stenosis)	Mechanical valve	
	Atrial or ventricular ectopic beats, isolated	Most arrhythmias (supraventricular arrhythmias)	Marfan or other HTAD syndrome without aortic dilatation	Systemic right ventricle with good or mildly decreased ventricular function	Severe systemic ventricular dysfunction (EF <30% or NYHA classes lll–IV)
		Turner syndrome without aortic dilatation	Aorta < 45 mm in bicuspid aortic valve pathology	Fontan circulation	A patient with Fontan circulation experiencing any medical complication
					Severe mitral stenosis
					Severe symptomatic aortic stenosis
					Systemic right ventricle with moderate or severely decreased ventricular function
			Repaired coarctation	Unrepaired cyanotic heart disease	
			Atrioventricular septal defect		
				Other complex heart disease	
				Moderate mitral stenosis	Severe aortic dilatation (>45 mm in Marfan syndrome or other HTAD, >50 mm in bicuspid aortic valve. Turner syndrome ASI >25 mm/m^2^, tetralogy of Fallot >50 mm)
				Severe asymptomatic aortic stenosis	
				Moderate aortic dilatation (40–45 mm in Marfan syndrome or other HTAD; 45–50 mm in bicuspid aortic valve. Turner syndrome ASI 20–25 mm/m^2^, tetralogy of Fallot <50 mm)	
					Vascular Ehlers–Danlos syndrome
					Severe (re)coarctation
				Ventricular tachycardia	

*ASI* aortic size index, *EF* ejection fraction, *HTAD* hereditary thoracic aortic disease, *NYHA* New York Heart Association

### The pregnancy heart team

The pregnancy heart team has been put forward in the new guidelines and evolved from the multidisciplinary management team of the 2011 guidelines. The pregnancy heart team focuses on women with a moderate or high risk of complications during pregnancy (mWHO II–III, III and IV), providing prepregnancy counselling and management during pregnancy and around delivery at an expert center. The minimum team requirements are a cardiologist, an obstetrician and an anesthetist, all with sufficient expertise in the management of high-risk pregnancies in women with cardiovascular diseases. Pregnant women with artificial heart valves should be cared for by the pregnancy heart team.

### Treatment of hypertension

Pregnant women with moderate to high risk for pre-eclampsia should start with 100–150 mg Aspirin per day at the beginning of week 12 and continue this medication until weeks 36–37.

All pregnant women with sustained elevation of blood pressure ≥150/95 mm Hg should receive antihypertensive treatment.

Pregnant women with sustained elevation of blood pressure >140/90 mm Hg should receive antihypertensive treatment in the presence of: pre-eclampsia, known arterial hypertension, arterial hypertension with diagnosed organ damage.

Methyldopa, labetalol, and calcium antagonists are recommended to treat arterial hypertension in pregnancy.

### Assisted reproductive therapy

Despite meeting patient wishes and expectations for reproduction in difficult settings, assisted reproduction entails additional risks on top of the underlying risk of pregnancy. For example, superovulation because of ovarian hyperstimulation (OHSS) leads to significant fluid shifts and an increased risk of thrombosis [7]. The risk of OHSS can be reduced by use of low-dose follicle-stimulating hormone (FSH) in combination with a gonadotropin-releasing hormone (GnRH) antagonist, and careful monitoring. Single embryo transfer is the preferred technique in women with heart disease because it is known that twin pregnancies are associated with significantly more complications than singleton pregnancies [8].

### Use of bromocriptine in peripartum cardiomyopathy (PPCM)

The EURObservational Research Programme international PPCM registry has provided important new information on PPCM [9]. Key predisposing factors include multiparity, African ethnicity, smoking, diabetes, pre-eclampsia, malnutrition, advanced age and teenage pregnancy. The cause of PPCM is uncertain but potential etiologies include inflammation and vascular damage. The biologically active 16-kDa prolactin and other factors, such as soluble fms-like tyrosine kinase 1 (sFlt1), may initiate and drive PPCM [10]. Based on these insights, bromocriptine (2.5 mg once daily) for at least 1 week may be considered in uncomplicated cases, whereas prolonged treatment (2.5 mg twice daily for 2 weeks, then 2.5 mg once daily for 6 weeks) may be considered in patients with ejection fraction (EF) <25% and/or cardiogenic shock. Bromocriptine treatment should be accompanied by anticoagulation with heparin, low molecular weight heparin (LMWH) or unfractionated heparin, at least in prophylactic dosages. Essential steps of treatment for patients with acute PPCM have been summarized as bromocriptine, oral heart failure therapies, anticoagulants, vasorelaxing agents, and diuretics (BOARD) [11].

### Advice on contraception and the termination of pregnancy in women with cardiac diseases

Women with a moderate or high risk of complications during pregnancy (mWHO II–III, III and IV) should be advised for a particular type of contraception with respect to the risk of pregnancy according to the mWHO classification (see above), which is able to estimate the risk with each method for a given medical condition. Advice is best provided by cardiologists with appropriate training or obstetricians and should be given at the time of menarche. An important rule is that unplanned pregnancy is particularly risky and has to be avoided. In the UK up to 30% of the first sexual intercourse occur before the age of 15 years [12] regardless of the presence of heart disease [13]. The key issues for a given contraceptive method are reliability and potential for complications, with thrombosis and infections being the most important; however, there are also advantages of hormonal contraception, such as control of menstruation, prevention of anemia, reduction of dysmenorrhea and prevention of hyperandrogenism.

### Further innovative contents of the new guidelines

Specific levels of surveillance for arrhythmia are provided with respect to the risk level for hemodynamic compromise at delivery.

New information on pharmacokinetics in pregnancy is given, including more detailed information on pharmacodynamics in animal experiments on all drugs.

The U.S. Food & Drug Administration (FDA) categories A–X as used for all drugs in 2011 have been replaced by descriptive risk summaries and preclinical safety data, including those for new drugs.

#### Novel messages of the new 2018 ESC guidelines for the management of cardiovascular diseases during pregnancy

- The use of the mWHO classification of maternal risk is recommended.
- The pregnancy heart team is introduced.
- Assisted reproductive therapy is defined.
- Bromocriptine is recommended for the treatment for peripartum cardiomyopathy (PPCM).
- Advice is given on contraception and the termination of pregnancy in women with cardiac diseases.
